# Comparative Analysis of Beta-Blockers Versus Calcium Channel Blockers in the Management of Chronic Stable Angina

**DOI:** 10.7759/cureus.108528

**Published:** 2026-05-08

**Authors:** Abdur Rehman, Tassawwar Iqbal, Saeeda Shahid, Manahil Zahra, Ahmed Ali, Nouman Zahid Bhutta, Muhammad Sanwal Abrar, Mateen Ahmed

**Affiliations:** 1 Cardiology, Punjab Institute of Cardiology, Lahore, PAK; 2 Cardiology, Major Shabbir Sharif Shaheed THQ, Kunjah, PAK; 3 General Medicine, Ali Medical Centre, Islamabad, PAK; 4 Medicine, Ayub Teaching Hospital, Abbottabad, PAK; 5 General Practice, Sadiq Health and Welfare Trust, Nalian, PAK; 6 Internal Medicine, Social Security Hospital, Gujrat, PAK; 7 Surgery, Shahida Islam Teaching Hospital, Lodhran, PAK

**Keywords:** beta-blockers, calcium channel blockers, chronic stable angina, coronary artery disease, exercise tolerance, nitrate use

## Abstract

Background: Chronic stable angina is a common manifestation of coronary artery disease that significantly affects quality of life. Beta-blockers and calcium channel blockers are widely used for symptom control; however, their comparative effectiveness remains an area of clinical interest.

Objective: This study aimed to compare the effectiveness of beta-blockers and calcium channel blockers in the management of chronic stable angina in terms of symptom control, exercise tolerance, and clinical outcomes.

Methodology: This comparative observational study was conducted at the Department of Cardiology, Punjab Institute of Cardiology, Lahore, Pakistan, from June 2025 to December 2025, and included 260 patients diagnosed with chronic stable angina.

Results: The mean age of the participants was 61.5 ± 9.3 years, with 148 (56.9%) males and 112 (43.1%) females. Beta-blockers achieved better heart rate control compared with calcium channel blockers (68.2 ± 7.5 bpm vs 74.6 ± 8.1 bpm, p < 0.001), with a greater proportion of patients achieving controlled heart rate (92 (70.8%) vs 54 (41.5%)). Patients receiving beta-blockers experienced fewer anginal episodes (2.1 ± 1.3 vs 2.8 ± 1.5, p = 0.002) and lower nitrate use (1.6 ± 1.1 vs 2.3 ± 1.4, p = 0.001). Exercise tolerance was also higher in the beta-blocker group (6.8 ± 1.4 metabolic equivalents (METs) vs 6.1 ± 1.6 METs, p = 0.003). Overall symptom improvement was greater with beta-blockers (72.5 ± 12.6% vs 65.3 ± 13.8%, p = 0.001), along with fewer hospital visits (1.4 ± 0.8 vs 1.9 ± 1.1, p = 0.002) and higher patient satisfaction scores (8.4 ± 1.1 vs 7.8 ± 1.3, p = 0.004).

Conclusion: Beta-blockers demonstrated superior outcomes compared with calcium channel blockers in controlling symptoms and improving functional capacity in patients with chronic stable angina.

## Introduction

Chronic stable angina is a common manifestation of coronary artery disease, characterized by typical chest pain resulting from myocardial ischemia caused by an imbalance between myocardial oxygen supply and demand [1]. It significantly affects quality of life and is associated with increased cardiovascular morbidity if left untreated [2]. The primary goals of treatment are symptom reduction, improvement in exercise tolerance, and prevention of adverse cardiovascular events. Pharmacological therapy mainly includes antianginal agents such as beta-blockers and calcium channel blockers [3]. These drug classes differ in their mechanisms of action, but both aim to reduce myocardial oxygen demand and improve coronary perfusion. Beta-blockers decrease heart rate, myocardial contractility, and blood pressure, thereby reducing myocardial oxygen consumption and prolonging diastole, which improves coronary filling [4]. They are considered first-line therapy, particularly in patients with a history of myocardial infarction or impaired left ventricular function [5]. In contrast, calcium channel blockers produce vasodilation and reduce afterload, with some agents also decreasing heart rate and myocardial contractility [6]. They are especially useful in patients who are unable to tolerate beta-blockers or have contraindications such as bronchial asthma [7].

Although both classes are effective in relieving anginal symptoms, their adverse effect profiles differ. Beta-blockers are commonly associated with bradycardia and fatigue, whereas calcium channel blockers may cause peripheral edema, hypotension, and headache [8]. Therefore, the choice of therapy is often influenced by individual patient characteristics and comorbid conditions [9]. Previous studies have shown that beta-blockers reduce the frequency of anginal episodes and improve exercise capacity [10]. Calcium channel blockers have also demonstrated strong efficacy in symptom control, particularly in vasospastic angina [11]. However, their comparative effectiveness in chronic stable angina remains an area of ongoing evaluation [12]. Optimizing symptom control, improving patient adherence, and maintaining functional status are important considerations in long-term management [13]. Despite the widespread clinical use of both drug classes, direct comparative data across different clinical settings remain limited [14].

Objective

This study aimed to evaluate the association between beta-blocker and calcium channel blocker monotherapy with symptom control, exercise tolerance, and clinical outcomes in patients with chronic stable angina.

## Materials and methods

Methodology

This comparative observational study was conducted at the Department of Cardiology, Punjab Institute of Cardiology, Lahore, Pakistan, from June 2025 to December 2025, and included 260 patients diagnosed with chronic stable angina. Patients aged ≥40 years with chronic stable angina, diagnosed based on clinical evaluation and ECG findings, were included in the study. Only patients receiving either beta-blockers or calcium channel blockers as monotherapy, and who had stable symptoms for at least three months prior to enrollment, were eligible. In addition, participants were required to be willing to participate and able to attend follow-up visits. Patients with unstable angina or recent myocardial infarction were excluded. Those receiving combination antianginal therapy (beta-blockers and calcium channel blockers), as well as patients with severe heart failure, significant valvular heart disease, or arrhythmias affecting heart rate control, were also excluded. Furthermore, patients with contraindications to either beta-blockers or calcium channel blockers were not included in the study.

Data collection

After obtaining informed consent, demographic and clinical data were recorded using a structured proforma. A detailed clinical assessment was performed, including history of anginal episodes, duration of disease, and comorbid conditions such as hypertension and diabetes mellitus. Patients were categorized into two groups based on the antianginal monotherapy already prescribed by the treating cardiologist as part of routine clinical care: the beta-blocker group and the calcium channel blocker group. Group allocation was not randomized. The choice of therapy was based on clinical judgment, patient profile, comorbid conditions, contraindications, resting heart rate, blood pressure, and treatment tolerance. To minimize misclassification bias, only patients maintained on the same monotherapy during the assessment period were included. This observational grouping was used to assess associations between treatment class and outcomes rather than to establish causality. Baseline investigations included ECG and echocardiography, where indicated. Clinical outcomes were assessed by evaluating the frequency of anginal attacks per week, use of sublingual nitrates, and exercise tolerance using a standardized grading scale. Heart rate and blood pressure were recorded at baseline and during follow-up. Patients were followed for symptom improvement and adverse effects. Treatment response was categorized as good, moderate, or poor based on reduction in anginal frequency and improvement in functional capacity. Patients were assessed after being on stable monotherapy for at least three months. Follow-up evaluations included symptom review, nitrate use, heart rate, blood pressure, exercise tolerance, adverse effects, and overall treatment response.

Statistical analysis

Data were entered into Microsoft Excel (Microsoft Corp., Redmond, WA, USA) and analyzed using IBM SPSS Statistics for Windows, Version 26.0 (Released 2018; IBM Corp., Armonk, NY, USA). Continuous variables were expressed as mean ± standard deviation, while categorical variables were presented as frequencies and percentages. Comparisons between the two groups were performed using the independent t-test for continuous variables and the chi-square test for categorical variables. A p-value < 0.05 was considered statistically significant.

## Results

A total of 260 patients were included, with a mean age of 61.5 ± 9.3 years. Most patients were aged 56-65 years (96; 36.9%), followed by those aged >65 years (92; 35.4%) and 40-55 years (72; 27.7%). There were 148 males (56.9%) and 112 females (43.1%). Diabetes mellitus was present in 104 patients (40.0%), while hypertension was present in 138 (53.1%). The mean duration of angina was 3.8 ± 1.6 years. Baseline heart rate was 82.4 ± 9.8 beats per minute, and mean systolic blood pressure was 138.6 ± 14.2 mmHg (Table 1).

**Table 1:** Baseline demographic and clinical characteristics (n = 260)

Variable	Category/mean ± SD	Total (n = 260)
Age (years)	61.5 ± 9.3	61.5 ± 9.3
Age group	40-55 years	72 (27.7%)
	56-65 years	96 (36.9%)
	>65 years	92 (35.4%)
Gender	Male	148 (56.9%)
	Female	112 (43.1%)
Diabetes mellitus	Yes	104 (40.0%)
	No	156 (60.0%)
Hypertension	Yes	138 (53.1%)
	No	122 (46.9%)
Duration of angina (years)	3.8 ± 1.6	3.8 ± 1.6
Baseline heart rate (bpm)	82.4 ± 9.8	82.4 ± 9.8
Baseline systolic blood pressure (mmHg)	138.6 ± 14.2	138.6 ± 14.2

Patients receiving beta-blockers had a lower mean heart rate (68.2 ± 7.5 bpm) compared with those receiving calcium channel blockers (74.6 ± 8.1 bpm, p < 0.001). Systolic blood pressure was also slightly lower in the beta-blocker group (126.4 ± 11.8 mmHg vs 129.7 ± 12.5 mmHg, p = 0.04), as was diastolic blood pressure (78.3 ± 7.2 mmHg vs 80.6 ± 7.9 mmHg, p = 0.03). Controlled heart rate (<70 bpm) was achieved in 92 patients (70.8%) in the beta-blocker group compared with 54 patients (41.5%) in the calcium channel blocker group (p < 0.001). Adverse effects were observed in 18 patients (13.8%) and 26 patients (20.0%), respectively (Table 2).

**Table 2:** Distribution of treatment groups and hemodynamic parameters BP: blood pressure, HR: heart rate.

Variable	Beta-blockers (n = 130)	Calcium channel blockers (n = 130)	p-value
Heart rate (bpm)	68.2 ± 7.5	74.6 ± 8.1	<0.001
Systolic blood pressure (mmHg)	126.4 ± 11.8	129.7 ± 12.5	0.04
Diastolic blood pressure (mmHg)	78.3 ± 7.2	80.6 ± 7.9	0.03
Patients with controlled heart rate (<70 bpm)	92 (70.8%)	54 (41.5%)	<0.001
Adverse effects	18 (13.8%)	26 (20.0%)	0.18

Beta-blockers demonstrated better symptom control, with fewer anginal episodes per week (2.1 ± 1.3 vs 2.8 ± 1.5, p = 0.002) and lower nitrate use (1.6 ± 1.1 vs 2.3 ± 1.4, p = 0.001). Exercise tolerance was also higher in the beta-blocker group (6.8 ± 1.4 metabolic equivalents (METs)) compared with the calcium channel blocker group (6.1 ± 1.6 METs, p = 0.003). A good treatment response was observed in 78 patients (60.0%) in the beta-blocker group compared with 59 patients (45.4%) in the calcium channel blocker group, while a poor response was seen in 14 (10.8%) versus 22 (16.9%) patients, respectively (Table 3).

**Table 3:** Anginal symptom control and functional outcomes

Variable	Beta-blockers (n = 130)	Calcium channel blockers (n = 130)	p-value
Anginal episodes/week	2.1 ± 1.3	2.8 ± 1.5	0.002
Nitrate use/week	1.6 ± 1.1	2.3 ± 1.4	0.001
Exercise tolerance (metabolic equivalents, METs)	6.8 ± 1.4	6.1 ± 1.6	0.003
Good response	78 (60.0%)	59 (45.4%)	0.02
Moderate response	38 (29.2%)	49 (37.7%)	
Poor response	14 (10.8%)	22 (16.9%)	

Overall symptom improvement was greater in patients receiving beta-blockers (72.5 ± 12.6%) compared with those receiving calcium channel blockers (65.3 ± 13.8%, p = 0.001). The mean number of hospital visits per year was lower in the beta-blocker group (1.4 ± 0.8 vs 1.9 ± 1.1, p = 0.002). Patient satisfaction scores were also higher with beta blockers (8.4 ± 1.1 vs 7.8 ± 1.3, p = 0.004). Drug compliance was slightly better in the beta-blocker group (110 patients; 84.6%) compared with the calcium channel blocker group (102 patients; 78.5%). The need for therapy change was lower in the beta blocker group (16 patients; 12.3% vs 27 patients; 20.8%, p = 0.05) (Table 4).

**Table 4:** Overall clinical outcomes and patient satisfaction

Variable	Beta-blockers (n = 130)	Calcium channel blockers (n = 130)	p-value
Symptom improvement (%)	72.5 ± 12.6	65.3 ± 13.8	0.001
Hospital visits (per year)	1.4 ± 0.8	1.9 ± 1.1	0.002
Patient satisfaction score	8.4 ± 1.1	7.8 ± 1.3	0.004
Drug compliance	110 (84.6%)	102 (78.5%)	0.21
Need for therapy change	16 (12.3%)	27 (20.8%)	0.05

## Discussion

Chronic stable angina is an important clinical manifestation of coronary artery disease, and its management is primarily focused on symptom control. The mean age of patients in this study was 61.5 ± 9.3 years, with a male predominance (56.9%). These findings are consistent with previous studies, which have reported a higher prevalence of angina in older adults and males. In addition, the frequent occurrence of comorbidities in this study population (diabetes mellitus 40.0% and hypertension 53.1%) is in agreement with established evidence linking these conditions to ischemic heart disease [15]. The present study demonstrated that beta-blockers provided better heart rate control (68.2 ± 7.5 bpm) compared with calcium channel blockers (74.6 ± 8.1 bpm), along with a higher proportion of patients achieving target heart rate (<70 bpm) (70.8% vs 41.5%). Similar findings have been reported in previous studies, which show greater reductions in heart rate with beta-blockers due to their negative chronotropic effects, thereby reducing myocardial oxygen demand [16]. Patients receiving beta-blockers also showed superior symptom control, with fewer anginal episodes per week (2.1 ± 1.3 vs 2.8 ± 1.5) and lower nitrate use (1.6 ± 1.1 vs 2.3 ± 1.4). Exercise tolerance was also higher in the beta-blocker group (6.8 ± 1.4 METs vs 6.1 ± 1.6 METs). These findings indicate better overall functional outcomes with beta-blockers and are consistent with previous reports demonstrating improved exercise capacity and reduced anginal frequency with their use [17]. The proportion of patients achieving a good treatment response was higher in the beta-blocker group (60.0%) compared with the calcium channel blocker group (45.4%), while poor response was less frequent (10.8% vs 16.9%). Enhanced symptomatic improvement with beta-blockers, particularly in patients with heart rate-dependent ischemia, has also been demonstrated in other studies [18].

In addition, overall symptom improvement was greater in patients receiving beta-blockers (72.5 ± 12.6%) compared with those receiving calcium channel blockers (65.3 ± 13.8%). Fewer hospital visits (1.4 ± 0.8 vs 1.9 ± 1.1) and higher patient satisfaction scores (8.4 ± 1.1 vs 7.8 ± 1.3) were also observed in the beta-blocker group. These findings suggest better clinical stability and greater perceived patient benefit, which is consistent with previous studies reporting improved quality of life and reduced healthcare utilization associated with effective heart rate control [19]. Although the incidence of adverse effects was slightly higher in the calcium channel blocker group (20.0% vs 13.8%), the difference was not statistically significant. Previous studies have also reported comparable tolerability between the two drug classes, with side effects largely dependent on individual patient characteristics and the specific agent used [20]. The need for therapy change was lower in the beta-blocker group (12.3% vs 20.8%), suggesting greater treatment sustainability. This aligns with earlier research indicating better long-term adherence and effectiveness of beta-blockers as first-line therapy in chronic stable angina. Overall, this study suggests that beta-blockers may be superior to calcium channel blockers in the management of chronic stable angina in terms of heart rate control, symptom reduction, and functional capacity. These findings are consistent with existing literature and support the use of beta-blockers as an effective first-line treatment option in this patient population.

However, certain limitations should be considered when interpreting these findings. The observational and non-randomized design introduces potential selection bias and confounding by indication, as treatment allocation was based on routine clinical judgment. Baseline comparability between treatment groups was not fully established, and no multivariable adjustment was performed to control for potential confounders. Several outcomes were subjective and were not assessed using validated instruments, which may increase the risk of measurement bias. In addition, treatment protocols were not standardized with respect to specific drug type, dose, or titration. The single-center design and relatively short follow-up period further limit the generalizability and long-term interpretability of the results.

## Conclusions

It is concluded that beta-blockers demonstrated better effectiveness compared with calcium channel blockers in the management of chronic stable angina, with improved heart rate control, reduced frequency of anginal episodes, lower nitrate use, and enhanced exercise tolerance. Beta-blocker use was associated with more favorable symptom control and functional outcomes than calcium channel blocker use in this cohort of patients with chronic stable angina. However, due to the observational, non-randomized design and other methodological limitations, no definitive conclusion regarding superiority can be made. Larger prospective studies with standardized treatment protocols and adjustment for confounding factors are required.
